# Maternal sepsis and factors associated with poor maternal outcomes in a tertiary hospital in Tigray, Ethiopia: a retrospective chart review

**DOI:** 10.1186/s12879-024-09075-9

**Published:** 2024-02-07

**Authors:** Bisrat Tesfay Abera, Hale Teka, Daniel Gebre, Tsega Gebremariam, Ephrem Berhe, Hagos Gidey, Birhane Amare, Rahel Kidanemariam, Marta Abrha Gebru, Fireweyni Tesfay, Yibrah Berhe Zelelow, Awol Yemane, Fanus Gebru, Ashenafi Tekle, Habtom Tadesse, Mohammedtahir Yahya, Ytbarek Tadesse, Hiluf Ebuy Abraha, Mussie Alemayehu, Mohamedawel Mohamedniguss Ebrahim

**Affiliations:** 1 Department of Internal Medicine, School of Medicine, Mekelle University, Box: 1871, Mekelle, Tigray, Ethiopia; 2 Department of Obstetrics and Gynecology, School of Medicine, Mekelle University, Mekelle, Tigray, Ethiopia; 3 Department of Midwifery, Ayder Comprehensive Specialized Hospital, Mekelle, Tigray, Ethiopia; 4 Department of Biostatistics, School of Public Health, Mekelle University, Mekelle, Tigray, Ethiopia; 5 Department of Reproductive Health, School of Public Health, Mekelle University, Mekelle, Tigray, Ethiopia; 6 University of South Carolina, Arnold School of Public Health, Columbia, South Carolina USA

**Keywords:** Maternal sepsis, Severe maternal outcomes, Associated factors, Tigray, Ethiopia

## Abstract

**Background:**

Maternal sepsis is the third leading cause of maternal death in the world. Women in resource-limited countries shoulder most of the burdens related to sepsis. Despite the growing risk associated with maternal sepsis, there are limited studies that have tried to assess the impact of maternal sepsis in resource-limited countries. The current study determined the outcomes of maternal sepsis and factors associated with having poor maternal outcomes.

**Methods:**

A facility-based retrospective cross-sectional study design was employed to assess the clinical presentation, maternal outcomes, and factors associated with maternal sepsis. The study was conducted in Ayder Comprehensive Specialized Hospital, Tigray, Ethiopia, from January 1, 2017, to December 31, 2021. Sociodemographic characteristics, clinical characteristics and outcomes of women with maternal sepsis were analyzed using a descriptive statistic. The association between dependent and independent variables was determined using multivariate logistic regression.

**Results:**

Among 27,350 live births, 298 mothers developed sepsis, giving a rate of 109 maternal sepsis for every 10,000 live births. There were 22 maternal deaths, giving rise to a case fatality rate of 7.4% and a maternal mortality ratio of 75 per 100,000 live births. Admission to the intensive care unit and use of mechanical ventilator were observed in 23.5% and 14.1% of the study participants, respectively. A fourth (24.2%) of the mothers were complicated with septic shock. Overall, 24.2% of women with maternal sepsis had severe maternal outcomes (SMO). Prolonged hospital stay, having parity of two and above, having the lung as the focus of infection, switchof antibiotics, and developing septic shock were significantly associated with SMO.

**Conclusions:**

This study revealed that maternal sepsis continues to cause significant morbidity and mortality in resource-limited settings; with a significant number of women experiencing death, intensive care unit admission, and intubation attributable to sepsis. The unavailability of recommended diagnostic modalities and management options has led to the grave outcomes observed in this study. To ward off the effects of infection during pregnancy, labor and postpartum period and to prevent progression to sepsis and septic shock in low-income countries, we recommend that concerted and meticulous efforts should be applied to build the diagnostic capacity of health facilities, to have effective infection prevention and control practice, and to avail recommended diagnostic and management options.

## Introduction

The World Health Organization (WHO) defines maternal sepsis as a life-threatening condition defined as organ dysfunction resulting from infection during pregnancy, childbirth, post-abortion, or postpartum period [1]. Maternal sepsis continues to cause significant mortality and morbidity worldwide. According to the WHO report, maternal sepsis is responsible for 10.7% of global maternal deaths annually [2]. Women in resource-limited countries shoulder most of the burdens related to sepsis. Lack of equipped diagnostic modalities, substandard management, lack of skilled birth attendants, poor infection prevention and control practice, and lack of access to clean water and sanitary materials contribute to a high rate of maternal sepsis and related mortality and morbidity in developing countries [3]. Although high-income countries have shown significant stride in decreasing maternal sepsis-related mortality and morbidity, low-income countries are lagging in implementing interventions set out by the WHO to lessen the impacts of maternal sepsis [4].

Diagnosing sepsis during pregnancy can be difficult due to physiological changes during pregnancy; and due to obstetric interventions during the peripartum period which alters the typical clinical manifestations. The absence of an effective protocol for diagnosis and management of sepsis in low-income countries leads to delay in diagnosis of sepsis; increasing occurrence of septic shock, admission to critical care, and the need for respiratory support [5].

The disparities in maternal sepsis care were highlighted in the 1-week prospective GLOSS study which assessed the rate of maternal infection and practice of care in 713 facilities from 52 countries [6]. The study highlighted the importance of implementing evidence-based practices to prevent, diagnose early, and manage maternal infections to minimize morbidity and mortality in health facilities. Undergoing baseline assessment to determine the impact of maternal sepsis in healthcare facilities located in low-income countries is important to improve the quality of care in women of reproductive age and to address health inequity across economic ladders of development. Despite the growing risk associated with maternal sepsis, there are limited studies that assessed the impact of maternal sepsis in resource-limited countries. There are no accessible published studies that determined the outcome of maternal sepsis in Ayder Comprehensive Specialized Hospital (ACSH). The current study determined the outcomes of maternal sepsis and factors associated with having poor maternal outcomes.

## Methods

### Study design

A facility-based retrospective study design was employed to assess the outcomes of maternal sepsis and to determine factors associated with severe maternal outcomes (SMO) in ACSH, Tigray, Ethiopia.

### Study setting

The study was carried out at ACSH, a tertiary care and teaching hospital located in the Tigray region of Ethiopia. With a catchment area of around eight million people, it is the largest referral hospital in the region. It also gives service to neighboring districts of the Afar and Amhara regions. There are 500 inpatient beds. Obstetrics and gynecology care are among the services delivered in the hospital. It has separate antenatal care clinics for low and high-risk patients. Currently, the hospital hosts an average of 5000 deliveries per year. The Obstetrics and Gynecology department is run by 20 consultants and 150 midwives The hospital offers both abortion and post-abortion care for spontaneous and medical abortion services. Moreover, ACSH is equipped to perform culture and sensitivity tests. From previously published reviews, the infamous triad namely obstetric hemorrhage, hypertensive disorders of pregnancy, and sepsis prevail as the main cause of maternal mortality in ACSH visa vis the Tigray region [7].

### Study period

The charts of all women who were diagnosed with maternal sepsis between January 1, 2017 and December 31, 2021 were reviewed.

### Study population

Records of all women who developed sepsis during pregnancy, childbirth, or within 42 days following spontaneous or medical abortionand/or postpartum period were included.

### Sample size and power

The charts of all women who were diagnosed with maternal sepsis between January 1, 2017 and December 31, 2021 were reviewed. The list of mothers was compiled from the maternity delivery logbook, the operating theater logbook, the emergency logbook, and the ICU admission registers. Due to the rarity of maternal sepsis, all cases were collected consecutively rather than employing a sampling technique. We computed the statistical power for a cohort of 298 mothers consecutively diagnosed with sepsis. Accordingly, with a sample size of 298, a significance level of 0.05, and a minimum effect size of 0.2, using single population proportion formula, the resulting calculated power was 0.93.

### Data collection tool and procedures

The data collection tool was developed after a thorough review of the literature, and the instrument was validated. A pilot test was then conducted to assess the tool’s usability and identify potential problems, leading to subsequent refinements. The Open Data Kit (ODK) tool was utilized for data collection. The data collection tool was used to collect information on sociodemographic characteristics, obstetric profile, comorbidity, clinical presentation, investigation, management, and outcomes of women with maternal sepsis. Trained medical doctors were responsible for collecting the data.

To guarantee the quality of the data, medical doctors involved in data collection underwent a one-week training. Moreover, throughout the data collection process, investigators conducted daily supervision to ensure data completeness, accuracy, and validity.

### Measurement

#### Sepsis

mothers who fulfilled the WHO criteria for maternal sepsis were included. The WHO defines sepsis as “organ dysfunction resulting from infection during pregnancy, childbirth, postabortion, or postpartum.

#### Septic shock

Mothers with sepsis who, despite adequate resuscitation, required vasopressors to maintain a mean arterial pressure of 65 mmHg.

#### Severe maternal outcomes (SMO)

A mother was labeled as having SMO if she had developed a maternal near-miss condition or if she died during the course of sepsis.

#### Maternal near-miss

Maternal near miss refers to a woman who nearly died but survived of complications during pregnancy, childbirth or within 42 days of termination of pregnancy [8].

### Data analysis

Data collected through ODK was exported into STATA version 16. Descriptive statistics was done to describe the distribution of the data. Frequencies and percentages were calculated to describe the result of categorical variables. The degree of association between the categorical variable of the independent variable and the dependent variable (SMO) was done using a Chi-squared test. Results were presented using a p-value. For continuous variables that were normally distributed, the results were described using mean (SD). Before proceeding to the final model to identify the factors associated with SMO, a bivariate analysis was performed to select a potential variable for the multivariate logistic regression analysis. Factors having a p-value of 0.25 or less in the bivariate were included in the multivariable logistic regression. Finally, independent variables associated with the SMO were considered and used in the final model. A P-value < 0.05 was considered statistically significant. Hosmer-Lemeshow goodness of fit model was done to assess the model’s fitness. Variation inflation factor (VIF) among the covariates was checked to assess the effect of collinearity, and variables with multicollinearity were dropped from the list.

## Results

### Sociodemographic characteristics

A total of 298 women participated in the study. The median age was 27 years (IQR, 8) with 36% of the participants being in the 25–34 age category. The majority of them were rural dwellers (60.7%). Close to 48% of the mothers were multiparous. Hypertensive disorders of pregnancy were observed in 20.1% of the study participants. Approximately 5% of the participants had comorbidities. Spontaneous vaginal delivery was the most common delivery mode (57.4%). Maternal sepsis was diagnosed during pregnancy, intrapartum and postpartum, and post-abortion in 17.1%, 69.1%, and 13.8% of the participants, respectively (Table 1).

### Rate of maternal sepsis, clinical presentation, focus of infection, and investigations

Among 27,350 live births, 298 mothers developed sepsis giving a rate of 109 maternal sepsis for every 10,000 live births. The commonest presenting symptom was fever [244(82%)] followed by abdominal pain [127 (42.6)] and foul-smelling vaginal discharge [118(39.6%). Almost all study participants [293(98.3%)] presented after 24 h of symptom onset. The most common focus of infection was pelvic (65.1%) followed by lung (14.8%) and wound site (9.7%).

Culture was done in only 7.1% of the study participants. Antibiotics were switched in 24.85 of the patients. It was empirically changed in 90.5% of them. Lactate measurement was done in none of the patients.

### Maternal outcomes

The percentage of women who were discharged improved was 87% while 3.7% of study participants went home against medical advice and/or were transferred to other health facilities. The maternal mortality rate was 75 per 100, 0000 deliveries. Admission to the ICU, use of mechanical ventilation (MV), and death were observed in 23.5%, and 14.1%, and 7.4% of the study participants, respectively. Septic shock was observed in 24.2% of the study participants. Only 8% of women with septic shock received norepinephrine as a vasopressor. Overall, 24.2% of the women had SMO.

**Table 1:** Sociodemographic characteristics, obstetric profile, and clinical characteristics of women with maternal sepsis at Ayder Comprehensive Specialized Hospital, Tigray, Ethiopia, 2017–2021 (n = 298)

Variable	Severe maternal outcomes		P-value
	Yes (1)	No (0)	
**Age**			
15–24	21(29.17)	90(39.82)	0.077
25–34	36(50.00)	110(48.67)	
35 and above	15(20.83)	26(11.50)	
Median (IQR)	27 (8)		
**Residence**			
Rural	50(69.44)	131(57.96)	0.082
Urban	22(30.56)	95(42.04)	
**Parity**			
One and below 1	28 (38.89)	127(56.19)	0.010
Two and above 2	44(61.11)	99(43.81)	
**ANC visit**			
Yes	30(41.67)	133(58.85)	0.011
No	42(58.33)	93(41.15)	
**Status at diagnosis of sepsis**			
Pregnant	19(26.39)	32(14.16)	
Post-abortion	10(13.89)	31(13.72)	0.050
Intrapartum and Postpartum	43(59.72)	163(72.12)	
**Hypertensive disorders of pregnancy**			
Yes	19(26.39)	41(18.14)	
No	53(73.61)	185(81.86)	0.129
**Other Obstetric morbidities**			
Yes	40(55.56)	131(57.96)	
No	32(44.44)	95(42.04)	0.719
**Chronic medical illnesses**			
Yes	9(12.50)	7(3.10)	
No	63(87.50)	219(96.90)	0.002
**Place of delivery**			
Health Facility	60(83.33)	200(88.50)	
Home	12(16.67)	26(11.50)	0.253
**Mode of delivery**			
SVD	48(66.67)	123(54.42)	
Non SVD (CS, instrumental or laparotomy)	24(33.33)	103(45.58)	0.067
**Duration of symptoms (in days)**			
≤ 1	14(10)	122(90)	
> 1	63(39)	99(61)	0.000
**Focus of infection**			
Lung	32(44.44)	12(5.31)	
Other	40(55.56)	214(94.69)	0.000
**Severity of sepsis**			
Sepsis alone	43(59.72)	217(96.02)	
Septic shock	29(40.28)	9(3.98)	0.000
**Site of acquisition of the infection (n = 263)**			
Community	37(54.41)	118(60.51)	
Hospital	31(45.59)	77(39.49)	0.379
**Switch of antibiotics**			
Yes	37(51.39)	37(16.37)	
No	35(48.61)	189(83.63)	0.000
**Surgical management of sepsis given**			
Yes	11(15.28)	49(21.68)	
No	61(84.72)	177(78.32)	0.238
**Was surgical procedure done before developing sepsis and while in sepsis for other purposes**			
Yes	29(40.28)	120(53.10)	
No	43(59.72)	106(46.90)	0.058
**Local complications of sepsis**			
Yes	8(11.11)	19(8.41)	
No	64(88.89)	207(91.59)	0.486
**Hospital Stay**			
≤ 7	17(23.61)	139(61.50)	
≥ 8	55(76.39)	87(38.50)	0.000

IQR = interquartile range

### Predictors of severe maternal outcomes

The results of multivariable logistic regression analysis revealed that women who stayed greater than 7 days in hospital during their sepsis management were 4 times more likely to develop SMO than those who stayed 7 days and below (AOR = 4.25,95% CI:1.52,11.87). Similarly, the odds of having SMO among mothers who had two and above parity was 2.4 times (AOR = 2.41,95% CI:1.04,5.60) higher compared to women who had a parity of one and below. An increased odds of having SMO was independently associated with having the lung as the focus of infection (AOR = 9.83,95% CI:3. 3.70,26.14) than their counterparts. The odds of developing SMO was 13 times (AOR = 24.19,95% CI:27.28,80.28) higher among women who had septic shock than among women who had sepsis alone. An increased odds of having SMO was also observed among women who had their antibiotics switched (AOR = 3.94, 95% CI: 1.60,9.69) than their counterparts (Table 2).

**Table 2:** Predictors of severe maternal outcomes in women with maternal sepsis at Ayder Comprehensive Specialized Hospital, Tigray, Ethiopia, 2017–2021 (n = 298)

Variable	Severe maternal outcomes		COR 95% CI	AOR 95% CI
	Yes	No		
	n(%)	n(%)		
**Hospital stays in days**				
7 days and below	17(23.61)	139(61.50)	1	1
> 7 days	55(76.39)	87(38.50)	5.17(2.82,9.48)	4.25(1.52,11.87) *
**ANC visit**				
Yes	30(41.67)	133(58.85)	1	1
No	42(58.33)	93(41.15)	2.00(1.17,3.43)	1.61(0.65,3.98)
**Hypertensive disorders of pregnancy**				
Yes	19(26.39)	41(18.14)	1.62(0.87,3.02)	1.51(0.56,4.08)
No	53(73.61)	185(81.86)	1	1
**Parity**				
One and below	28(38.89)	127(56.19)	1	1
Two and above	44(61.11)	99(43.81)	2.01(1.17,3.46)	2.41(1.04,5.60) *
**Presence of chronic medical illness**				
Yes	9(12.50)	7(3.10)	4.46(1.60, 12.48)	3.16(0.48,20.63)
No	63(87.50)	219(96.90)	1	1
**Mode of delivery**				
SVD	48(66.67)	123(54.42)	1.67(0.96,2.91)	1.67(0.67,4.18)
Non SVD (CS, instrumental or laparotomy)	24(33.33)	103(45.58)	1	1
**Focus of infection**				
Lung	32(44.44)	12(5.31)	14.26(6.77,30.04)	9.83(3.70,26.14) *
Others*	40(55.56)	214(94.69)	1	1
**Severity of sepsis**				
Septic shock	29(40.28)	9(3.98)	16.26(7.19,36.78)	24.19(7.28,80.28) *
Sepsis alone	43(59.72)	217(96.02)	1	1
**Switch of antibiotics**				
Yes	37(51.39)	37(16.37)	5.4(3.02,9.65)	3.94(1.60,9.69) *
No	35(48.61)	189(83.63)	1	1
**Status of sepsis diagnosis**				
Pregnancy	19(26.39)	32(14.16)	2.25(1.16,4.35)	2.01(0.64,6.35)
Post abortion	10(13.89)	31(13.72)	1.22(0.56,2.68)	2.38(0.66,8.44)
Intrapartum and Postpartum	43(59.72)	163(72.12)	1	1
**Site of infection acquired**				
Community	37(54.41)	118(60.51)	1	1
Hospital	31(45.59)	77(39.49)	1.28(0.73,2.24)	0.71(0.26,1.90)
**Residence**				
Urban	22(30.56)	95(42.04)	0.61(0.34,1.06)	1.37(0.56,3.33)
Rural	50(69.44)	131(57.96)	1	1
**Delivery place**				
Home	12(16.67)	26(11.50)	1.54(0.73,3.23)	1.21(0.35,4.17)
Health facilities	60(83.33)	200(88.50)	1	1

*Significant at p-value < 0.05

## Discussions

This hospital-based cross-sectional study demonstrated that maternal sepsis was associated with grave maternal outcomes. In the present study, the prevalence of maternal sepsis was 109 per 100,000 live births. The case fatality rate was 7.4% while maternal mortality associated with maternal sepsis was 75 per 100,000 live births. Factors related to obstetric profile, clinical presentation, focus of infection, and unavailability of diagnostic tests contributed to the poor outcome observed in this study. The results of this study reckon that maternal sepsis continues to be an alarming source of mortality and morbidity in low-income countries. This calls for a concerted effort from responsible stakeholders to promptly implement policies and guidelines of maternal sepsis management and avail important diagnostic tools.

The rate of maternal sepsis in the present study exceeded maternal sepsis rate reported from China which stands at 1.7 per 1000 deliveries [9]. Similar to our finding, maternal mortality in other resource-limited regions remains high. A study from Northern India reported a maternal sepsis rate of 16.5 per 10,000 mortalities which is lower than the current study found [10]. The difference emanates from the parameters both studies used to define sepsis. While our study used the 2016 WHO definition of sepsis, the Indian study used the SIRS criteria to diagnose maternal sepsis, which would have overestimated the rate of maternal sepsis.

The maternal mortality rate observed in our study is also higher than reports from high-income countries. Baueret al. found that maternal Sepsis claimed one woman per 105,384 deliveries in the United States of America (USA) [11]. These findings attest to the prevailing disparities in obstetric care delivery between low-income and high-income countries.

Furthermore, the current study revealed that the rate of admission to the ICU was high compared to studies carried out in India and developed countries such as Ireland, which reported that 7% and 2.6% of women with sepsis needed ICU admission, respectively [10, 12]. Although the percentage of women who needed MV support was lower than in an Indian study where 54% of women with sepsis needed MV, the percentage of women who needed MV in our study was high at 14.1% [10]. Recent advances in maternal sepsis diagnostics and management observed in high-income countries are lacking in developing countries; this is backed by our study where lactate measurement and culture were lacking. Lack of equipped maternal care and a dearth of diagnostics are the defining elements of health systems in low-income countries [13]. Failing to improve women’s health in developing countries results in significant reproductivity and economic loss. Improving the health of women is an indirect measurement of the overall quality of healthcare provision in a given society [14]. Late presentation is another factor that led to the increased rate of ICU admission and use of MV in our study. Ensuing management as early as possible is the single most important intervention to salvage individuals with sepsis [15]. Late presentation and lack of appropriate intervention are responsible for organ dysfunction that requires admission to the ICU and use of MV; and ultimately lead to death [5]. The probability of developing septic shock in women included in the study (12.8%) was also higher than what was witnessed in tertiary care in China where the incidence of septic shock was 10.5% [98]. Despite having well-equipped maternal care in developed countries, studies from the United Kingdom (UK) and the US revealed that the incidence of septic shock also remains at a high rate and continues to be a significant burden to the healthcare. This is due to a recent influx of maternal infections attributed to Group A β-hemolytic streptococcus bacteria [16]. Septic shock needs prompt intervention with an early start of antibiotics, vasopressors, and strict monitoring, which are largely lacking in resource-limited settings. Unable to diligently treat septic shock leads to high morbidity and mortality [15].

In the current study, maternal sepsis claimed the lives of 7.4% of the women included in the study. This is > 5x higher than what was reported by Acosta CDet al. who assessed outcomes of maternal sepsis in the UK by undergoing a case-control study which revealed a 1.4% death rate [16]. Despite witnessing a surge in maternal sepsis, high-income countries are better armed to treat the disease while low-income countries continue to suffer from underdiagnosis and under treatment highlighting an existing disparity in maternal health. In addition, women in low-income countries suffer from infectious comorbidities such as Human immunodeficiency virus and tuberculosis that contribute to undesirable outcomes in these settings [17].

Different factors were identified as having a significant association with SMO. Women who stayed for more than seven days in the hospital were at an increased risk of developing SMO. A longer period of hospital stay is associated with failure to respond to antibiotics and other management modalities, and patients are at an increased risk of hospital-acquired complications that significantly contribute to SMO [18]. Having a parity of two and above was another factor which was significantly associated with the occurrence of SMO. The risk of having obstetric comorbidities, including sepsis, increases with parity, which imparts a cumulative negative effect on the survival and overall health of mothers [19].

Women who had the lung as the focus of infection were also at significant risk of developing SMO. Our finding supports a study done by Acosta CD et al. which revealed that women with pneumonia are at an increased risk of poor maternal outcomes [20]. This group of women receive MV more often than those who have a non-respiratory focus of infection. Using MV leads to a longer hospital stay and ventilator-associated pneumonia, which further increases the risk of poor maternal outcomes [21]. The lungs are also a primary site for hospital-acquired infections and resistant microorganisms [22].

Determining the level of lactate is one of the bundles of sepsis management recommended in the diagnosis of septic shock and the monitoring of response [23]. There was no single determination of lactate level in our study as the hospital lacks this important measurement. This results in a delay in diagnosis of sepsis and septic shock which in turn hampers early institution of appropriate intervention. Septic shock is also associated with multi-organ failure and needs a high level of care, but hospital settings in resource-limited areas are at a disadvantage as they are not ready to deliver advanced care to such patients [24]. Not diagnosing sepsis and septic shock early has a devastating impact on the lives of women with maternal sepsis [25]. Our study revealed that developing septic shock was significantly associated with SMO. Hence, availing lactate measurement is of paramount importance and should be a top priority for the hospital and other stakeholders. Resource-limited countries also suffer from inadequacy of vasopressors. Despite norepinephrine being the recommended vasopressor in patients with sepsis, only six out of the 38 women who developed septic shock received norepinephrine in our study. Dopamine and epinephrine were the commonly prescribed vasopressors. These drugs are associated with the development of side effects such as arrhythmias which have a negative impact on the survival of women with septic shock. Our findings reveal that our hospital, despite being the only tertiary care and the final destination hospital in the region, lacks the necessary diagnostics and management modalities for septic shock. Therefore, the hospital should work towards improving the care of patients with sepsis by providing access to recommended investigations and treatment. International stakeholders which strive to improve the health of mothers should also help in arming health facilities in resource-limited countries with the necessary diagnostic and management modalities.

Finally, women whose antibiotics were switched during the course of sepsis management fared worse than those who did not. Culture was done in only 7.1% of the study participants. All samples were sent after the initiation of antibiotics. These scenarios create a fertile ground for the selection of drug-resistant microorganisms and a low rate of response to antimicrobials; causing immense mortality and morbidity [26]. This calls for a change of practice at the hospital level, and stakeholders should invest in equipping health facilities to better diagnose and manage maternal sepsis.

### Strengths and limitations of the study

The study explored five-year data to determine outcomes of maternal sepsis and its associated factors. It tried to assess the burden of maternal sepsis in resource-limited settings. However, it has its drawbacks. Data on sociodemographic and reproductive characteristics, timing of abortion, occupation, educational status, economic status, and marital status were missing from most records. Errors inherent to retrospective data collection are also possible. Therefore, the need for robust data to identify risk factors and the practice of maternal sepsis management among healthcare workers is underscored.

## Conclusions

The study showed that maternal sepsis is a source of significant morbidity and mortality in resource-limited settings with a significant number of women experiencing ICU admission, intubation, and death attributable to sepsis. Prolonged hospital stay, having parity more than one, having the lung as the focus of infection, the need to switch another antibiotics, and developing septic shock were significantly associated with poor maternal outcomes. The unavailability of the recommended diagnostic modalities and management options have led to grave outcomes observed in this study. The results of this study have significant implications for health policy and patient care. To achieve both national and international goals such as a target of less than 70 maternal deaths per 100,000 live births by 2030, practical steps should be taken to address most common causes of maternal death such as obstetric sepsis. To ward off the effects of infection during pregnancy, delivery, and postpartum, and to prevent progression of the infection to sepsis and septic shock, we recommend that concerted efforts must be applied to build the diagnostic capacity of health facilities; the practice in infection prevention and control should be improved; healthcare practitioners should stick to guidelines when they manage mothers with maternal sepsis; and recommended management options should be availed in low-income countries.

## Data Availability

All data relevant to the study are included in the manuscript or can be shared upon request. Data can be requested from the corresponding author (Dr. Bisrat Tesfay Abera; Email: bis9live@gmail.com).

## Abbreviations

ACSH: Ayder Comprehensive Specialized Hospital
ICU: Intensive care unit
IRB: Institutional Review Board
MV: Mechanical Ventilation
SMO: Severe maternal outcomes
WHO: World Health Organization
